# Optical Characterization of Lorentz Force Based CMOS-MEMS Magnetic Field Sensor

**DOI:** 10.3390/s150818256

**Published:** 2015-07-27

**Authors:** John Ojur Dennis, Farooq Ahmad, M. Haris Bin Md Khir, Nor Hisham Bin Hamid

**Affiliations:** 1 Department of Fundamental and Applied Sciences, Universiti Teknologi PETRONAS, Bandar Seri Iskandar 32610 Tronoh, Perak Darul Ridzuan, Malaysia; 2 Department of Electrical and Electronic Engineering, Universiti Teknologi PETRONAS, Bandar Seri Iskandar 32610 Tronoh, Perak Darul Ridzuan, Malaysia; E-Mails: harisk@petronas.com.my (M.H.B.M.K.); hishmid@petronas.com.my (N.H.B.H.)

**Keywords:** CMOS-MEMS, Lorentz force, optical characterization, magnetic sensor, optical sensing, resonator

## Abstract

Magnetic field sensors are becoming an essential part of everyday life due to the improvements in their sensitivities and resolutions, while at the same time they have become compact, smaller in size and economical. In the work presented herein a Lorentz force based CMOS-MEMS magnetic field sensor is designed, fabricated and optically characterized. The sensor is fabricated by using CMOS thin layers and dry post micromachining is used to release the device structure and finally the sensor chip is packaged in DIP. The sensor consists of a shuttle which is designed to resonate in the lateral direction (first mode of resonance). In the presence of an external magnetic field, the Lorentz force actuates the shuttle in the lateral direction and the amplitude of resonance is measured using an optical method. The differential change in the amplitude of the resonating shuttle shows the strength of the external magnetic field. The resonance frequency of the shuttle is determined to be 8164 Hz experimentally and from the resonance curve, the quality factor and damping ratio are obtained. In an open environment, the quality factor and damping ratio are found to be 51.34 and 0.00973 respectively. The sensitivity of the sensor is determined in static mode to be 0.034 µm/mT when a current of 10 mA passes through the shuttle, while it is found to be higher at resonance with a value of 1.35 µm/mT at 8 mA current. Finally, the resolution of the sensor is found to be 370.37 µT.

## 1. Introduction

Nowadays magnetic field sensors are used in many different applications such as automotive, hydrocarbon exploration, navigation systems, mineral prospecting, nondestructive testing, biological field and satellite commutation [1,2]. Many researchers have successfully introduced magnetic field sensors based on the existing technologies using different physical principles, such as superconducting, Hall effect, magnetoresistivity and electromagnetic induction [3,4,5,6]. However, these sensors have drawbacks such as bulky size, high power consumption, requiring special techniques for their fabrication and operation [2]. Based on the requirements of future markets such as telecommunication, automotive industry and consumer electronics products, magnetic sensors should be low cost, small in size, and consume low power with better sensitivity and resolution.

Micro-Electro-Mechanical Systems (MEMS) technologies offer an attractive solution and many researchers have demonstrated magnetic field sensors based on MEMS technologies [7,8]. Bahreyni and Shafai developed a magnetic field sensor based on shifts in resonance frequency. This sensor has a sensitivity of 69.9 Hz/T and a resolution of 217 nT, but its output is highly temperature dependent [9]. Magnetic field sensors based on MEMS technologies often use the resonance principle and the change in resonance (frequency or amplitude) is sensed through piezoresistive [10,11], capacitive [12,13] or optical sensing methods [14,15]. The MEMS magnetic field sensors that rely on magnetic materials [16] have the problem of hysteresis and they need special techniques for the deposition of magnetic materials that increases their fabrication cost. The solution to these problems are Lorentz force-based actuated MEMS magnetic field sensors [17]. However, Lorentz force based complementary-metal-oxide-semiconductor (CMOS) compatible MEMS magnetic field sensors provide [18,19] not only the opportunity for low cost, low power consumption, lack of hysteresis and small size devices, but also devices with better sensitivity and resolution.

This article reports a MEMS based Lorentz force magnetic field sensor fabricated from CMOS thin layers having wide dynamic range and benefits from its cost effective CMOS batch fabrication. To avoid all electromagnetic interfering signals, an optical characterization method is implemented to investigate the resonance frequency, quality factor, damping ratio, sensitivity and resolution of the CMOS-MEMS magnetic field sensor. The methodology for the variation of external magnetic field in the case of the packaged sensor chip during the optical characterization is also highlighted. The article is divided into four sections: the first section briefly introduces the magnetic field sensors by highlighting the issues associated with them and the contribution of this article. The second section briefly describes the working principle and modeling of the sensor. Section 3 explains the fabrication of the sensor and in Section 4, the optical characterization setup is covered comprehensibly and the experimental results obtained from the prototype are finally discussed.

## 2. Working Principle and Modeling of the Sensor

**Figure 1:**
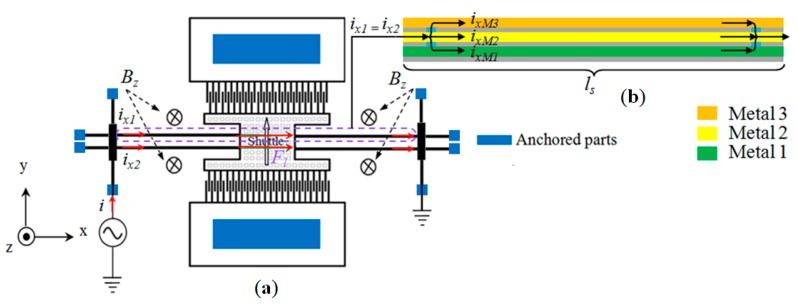
(**a**) Schematic of shuttle based magnetic field sensor in lateral mode; and (**b**) Cross-sectional view of the beam along the length.

The sensor consists of a resonating shuttle, which is the core part of the sensor. It connects its different parts to each other, both mechanically and electrically. It is anchored to stationary parts of the sensor by four long beams, as shown in Figure 1. In order to have the first mode of vibration of the shuttle in the *y* direction, long beams are designed to be much stiffer in the *z* direction than the *y* direction. The operating principle of the sensor is based on the differential change in the amplitude of the shuttle and this differential change can be sensed by two methods; optical and differential capacitance using comb fingers. The device can be actuated in static mode using a constant current or driven at its resonance frequency or any other desired frequency when an alternating current (ac) is passed through the shuttle beams with external magnetic field lines perpendicular to the current direction. The differential change in the amplitude of the shuttle represents the strength of the external magnetic field. The input current *i* to the shuttle enters the beams and divides into two parts *i_x1_* and *i_x2_* as shown in Figure 1. Each current then further divides into three paths, *i_xM3_*, *i_xM2_* and *i_xM1_*, following the three metal layers: metals 1, 2 and 3 as shown in the cross-sectional view across the length of the beam in Figure 1.

### Electromagnetic Actuation

The sensor is driven at its resonance frequency in the *y* direction by passing ac current with frequency equal to the fundamental (first mode of resonance) frequency of the shuttle. When the device is exposed to an external magnetic field, a Lorentz force (*F_l_*) is generated according to Equation (1):

(1) $$F_{l}=2n_{metal}iB_{z}l_{s}\sin\phi$$

(2) $$i=i_{x1}+i_{x2}$$

(3) $$i_{x1}=i_{x2}=i_{xM3}+i_{xM2}+i_{xM1}$$

where *n_metal_* is the number of metal layers, *B_z_* is the magnetic flux density in *z*-direction, *l_s_* is the sensor length (shuttle and beams), *ϕ* is the angle between the magnetic field lines and the current [20]. The differential change in amplitude *∆A* under electromagnetic actuation is given by Equation (4).

(4) ΔA=2nmetali(ΔBz)lssinϕm(ωn2−ωd2)2+(ωdγ)2

and the sensitivity is given by:

(5) ΔAΔBz=2nmetalilssinϕm(ωn2−ωd2)2+(ωdγ)2

where the *∆B_z_* is the differential change in the strength of the external magnetic field, *m* is the mass of shuttle, *ω_n_* and *ω_d_* are the natural and drive frequencies of the shuttle and *γ* is the damping constant. The relation among damping ratio *ξ*, natural frequency *ω_n_* and quality factor *Q* is shown in Equation (6):

(6) ξ=γ2ωn=12Q

The determination of quality factor from the resonance behaviour is given in Equation (7):

(7) $$Q=\frac{f_{r}}{\Delta f}$$

where *f_r_* is the resonance frequency and *∆f* is the frequency bandwidth.

## 3. Fabrication of the CMOS-MEMS Magnetic Sensor

A CMOS-MEMS technology is used to fabricate the shuttle based magnetic sensor. The sensor’s geometric parameters are listed in Table 1.

**Table 1:** Geometric parameters of the shuttle based magnetic field sensor.

Parameters	Symbol	Value	Unit
Sensor length	*l_s_*	755	µm
Long beam thickness	*t_b_*	~5	µm
Long beam width	*w_b_*	3	µm
Long beam length	*l_b_*	300	µm
Shuttle thickness	*t_shuttle_*	~5	µm
Shuttle width	*w_shuttle_*	100	µm
Shuttle length	*l_shuttle_*	155	µm
Shuttle finger thickness	*t_finger_*	~5	µm
Shuttle finger width	*w_finger_*	3	µm
Shuttle finger length	*l_finger_*	60	µm
Stator finger thickness	*t_stator,fing_*	~5	µm
Stator finger width	*w_stator,fing_*	3	µm
Stator finger length	*l_stator,fing_*	60	µm
Etch hole size	*A_etch,hole_*	10 × 10	µm
Mass of the shuttle	*m_shuttle_*	541.29 × 10^−12^	µm

**Figure 2:**
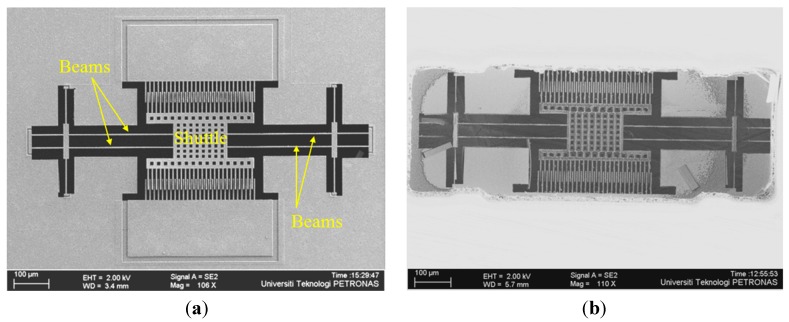
FESEM image of fully released device; (**a**) Front side view; (**b**) Back side view.

The fabrication of the device was completed at the MIMOS Bhd company (Kuala Lumpur, Malaysia) using three metal and two poly (3M, 2P) 0.35 µm CMOS technology. To release the MEMS device, dry post micromachining is carried out using a Tegal SS110A DRIE machine at MIMOS Bhd. A detailed description of the release process is given in [21]. The first step was thinning the wafer from 725 µm to 400 µm using a DISCO DFG 840 wafer back grinder machine. In the second step, Si was selectively etched from the backside up to ~360 µm depth. As a third step, top metal 3 was used as a mask during front side SiO_2_ and Si etching [22]. Finally, a fully released shuttle based magnetic field sensor is obtained as shown in Figure 2 with (a) front side and (b) back side view.

## 4. Optical Characterization Setup

The shuttle-based magnetic field sensor characterization setup for the lateral motion of shuttle comprises of standard optical and electrical instruments such as a Leica DM 12,000 optical microscope, AM2111 Dino-Lite digital microscope, and Agilent 33,522 function generator, respectively and a fine calibrated stainless steel ruler. This optical characterization set-up is based on the double magnification concept; one magnification through the Leica DM 12,000 optical microscope and second magnification through the Dino-Lite digital microscope. This technique is good for static and low frequency (~10 Hz) measurements, but equally good for high frequency (~10 kHz) measurements by, for example, measuring the amplitude of the shuttle at resonance.

### 4.1. Methodology for the Variation of the External Magnetic Field

**Figure 3:**
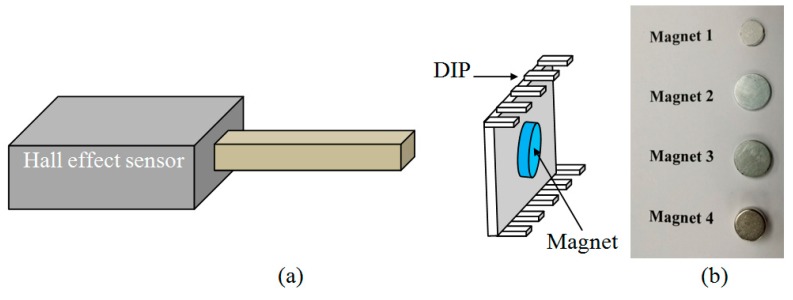
(**a**) Schematic of magnetic field measurement across the DIP with Hall effect sensor; (**b**) Magnets of different strengths used in the measurement.

**Figure 4:**
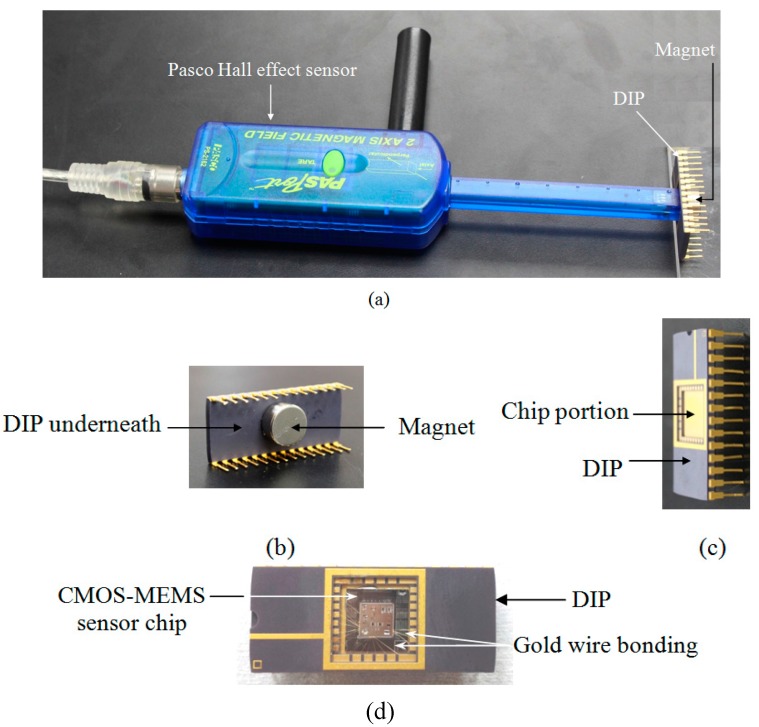
(**a**) Experimental setup to measure the strength of magnets placed under the DIP with Hall effect sensor; (**b**) back side of DIP showing attached magnet; (**c**) Front view of the DIP showing the die slot where the tip of the Hall effect sensor is placed; (**d**) Packaged CMOS-MEMS sensor chip.

First, the strengths of different magnets are accurately determined using standard Hall effect magnetic sensor as shown in schematic diagram in Figure 3a. The different magnets (number 1, 2, 3, 4) as shown in Figure 3b are placed underneath the Dual Inline Package (DIP) as shown in Figure 4b, one at a time, and its strength measured. The MEMS device is bonded on one side of the DIP package while permanent magnets of different strengths are placed underneath the DIP package. The packaged CMOS-MEMS sensor chip is shown in Figure 4d. The experimental setup for the measurement of strengths of magnets 1, 2, 3 and 4 is shown in Figure 4a.

### 4.2. Sensitivity Measurement in Static Mode

**Figure 5:**
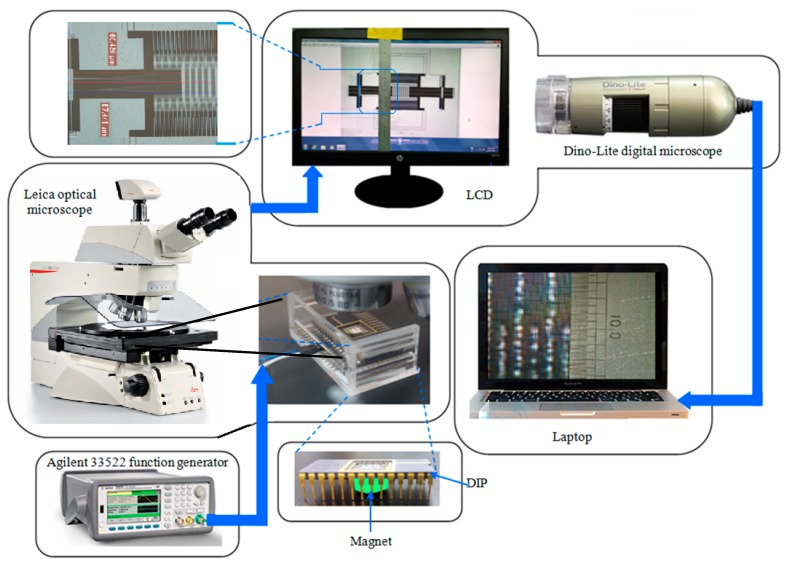
Schematic of the optical characterization set-up with the double magnification method.

The schematic diagram of the optical characterization set up is shown in Figure 5. The Leica optical microscope is the main heart of this technique. The MEMS device whose displacement is to be measured is placed under the optical microscope and the magnified image of moving part of the MEMS device is observed on the LCD monitor. There is a stainless steel ruler on the monitor which has the portion of marks calibrated up to micrometer level. The portion of image where displacement is to be measured is placed to coincide with the scale and a Dino-Lite digital microscope interfaced with a laptop computer is focused on that coinciding portion. Finally, the movement of the doubly magnified image along with the scale is observed and recorded on the laptop computer.

### 4.3. Sensitivity Measurement at Resonance Frequency

**Figure 6:**
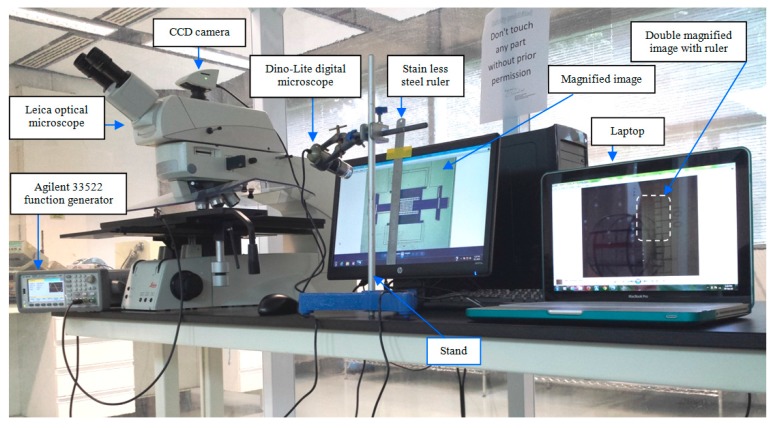
Experimental setup of the optical characterization with the double magnification method.

**Figure 7:**
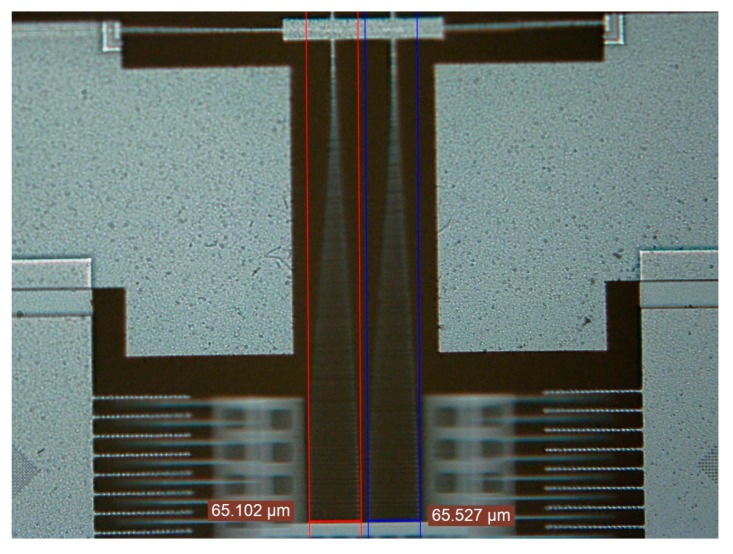
Resonance amplitude measurement under the microscope.

The schematic shown in Figure 5 and the actual experimental setup shown in Figure 6 are also used to measure the sensitivity of the shuttle based magnetic field sensor at its resonance frequency. Permanent magnets are placed underneath the DIP to obtain a magnetic field in the *z*-direction. At resonance, the differential change in the amplitude of the shuttle for different values of magnetic fields gives the sensitivity of the device. First, magnet 1 is placed underneath the DIP and the amplitude of the shuttle at its resonance frequency is measured using the Leica optical microscope as shown in Figure 7. Magnet 1 is then replaced by Magnet 2 and the measurement repeated. This is similarly repeated for the rest of the Magnets 3 and 4. The differential change in the amplitude of the resonating shuttle at different values of the magnetic fields is recorded and from these values, the sensitivity of the sensor is deduced.

## 5. Experimental Results and Discussion

The shuttle based magnetic field sensor is optically characterized in terms of resonance frequency, quality factor, sensitivity measurement in static mode as well as at resonance frequency and resolution measurement using the setup described in Section 4.

### 5.1. Shuttle Resonance Frequency, Quality Factor and Damping Ratio Measurements

**Figure 8:**
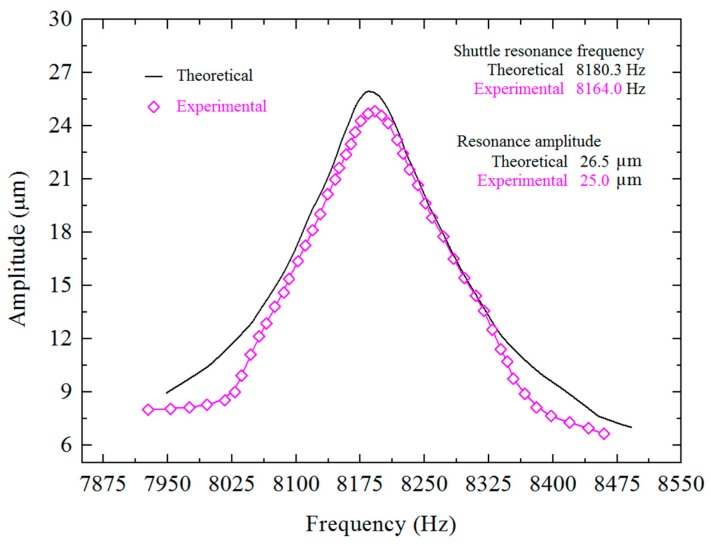
Resonance frequency of the shuttle.

The resonance frequency of the shuttle is determined using a 20.5 mT magnetic field underneath the DIP and applying an input signal of 2 Vpp and sweeping the frequency close to the theoretically calculated resonance frequency. The maximum amplitude of the vibration is then measured at the resonance frequency under the optical microscope. The experimentally determined resonance frequency is found to be 8.164 kHz and the corresponding amplitude is 25 µm. A comparison of theoretical and experimental resonance frequencies is shown in Figure 8 as well as in Table 2, indicating close agreement with 0.2% difference.

**Table 2:** Comparison of resonance frequencies, quality factors and damping ratios of the shuttle.

Parameters	Theoretical	Experimental	Percentage Difference
Resonance frequency	8180.3	8164.0	0.2%
Quality factor	53.6	51.34	4.2%
Damping ratio	0.00932	0.00973	4.3%

The 0.2% difference between the two values may be attributed to some minute amount of Si left underneath the CMOS layers of the shuttle during post-micromachining. Controlling the thickness of Si underneath the CMOS thin films during post-micromachining of MPW chips is very difficult and that is why up to 5% variation is considered normal [23]. The chips placed at the center of the carrier wafer have different Si thicknesses compared to those placed at the corners.

From the resonance curve of the shuttle as shown in Figure 8, the frequency band width Δ*f* is found to be 159 Hz, while *f*_2_ and *f*_1_ are 8240 Hz and 8081 Hz, respectively, and by using Equation (7), the quality factor is found to be 51.34. Equation (6) is used to determine the device’s damping ratio of 0.00973. The discrepancies between the experimental and theoretical values of quality factor and damping ratio are due to the fabrication tolerances. This value of the damping ratio includes intrinsic and extrinsic damping which can be minimized by vacuum packaging and the quality factor can thus be enhanced.

### 5.2. Calibration of the External Magnetic Field

To measure the sensitivity of CMOS-MEMS magnetic field sensor in lateral mode, the differential changes in amplitude corresponding to the different values of external magnetic fields are measured. The variation of external magnetic field is achieved by using permanent magnets of different strengths placed under the DIP and accurately calibrated as described in Section 4.1 with the help of a standard Hall effect magnetic sensor. The measurement results are shown in Table 3.

**Table 3:** Experimentally measured magnetic flux density.

Permanent Magnets	Magnetic Flux Density (mT)
Magnet 1	12.6
Magnet 2	20.5
Magnet 3	23.4
Magnet 1 + 2	30
Magnet 1 + 3	48.3
Magnet 2 + 3	57
Magnet 4	82

### 5.3. Sensitivity and Resolution Measurement

**Figure 9:**
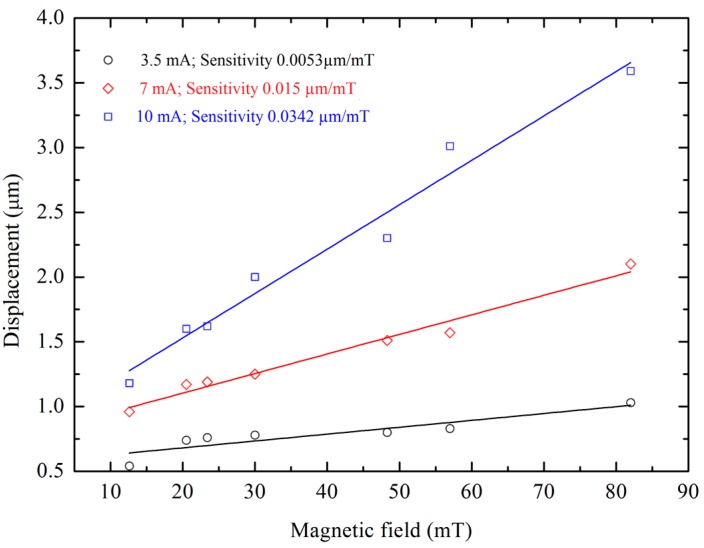
Magnetic field *vs*. shuttle displacement.

**Figure 10:**
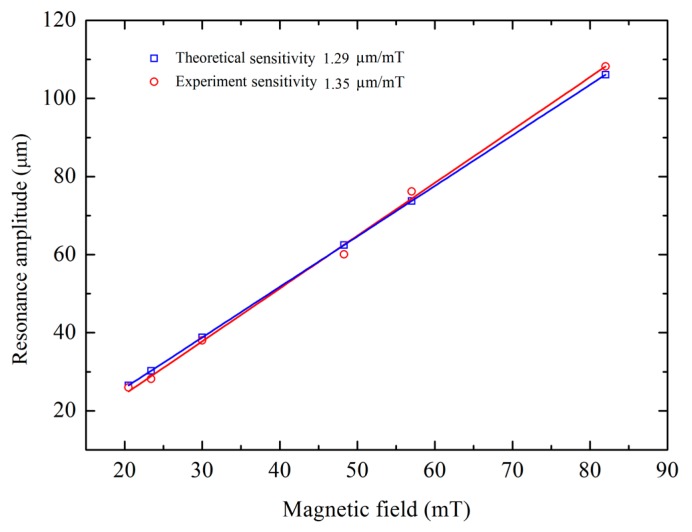
Magnetic field *vs*. shuttle amplitude at resonance.

To measure the sensitivity of the magnetic sensor in static mode, the magnetic field in the *z*-direction is varied by changing the magnets underneath the DIP while keeping the current through the shuttle constant at 3.5 mA and the corresponding displacement of the comb fingers on the shuttle are recorded. A similar procedure is followed for the current of 7 mA and 10 mA and all these trends are plotted in Figure 9 indicating increasing sensitivity as a function of current with the highest sensitivity of 34.27 nm/mT at 10 mA current. The 10 mA current is suitable for the measurement of magnetic fields in the microTesla range, the 7 mA current for the magnetic fields in the range of miliTesla and the 3 mA current for measurement in the Tesla range. Therefore, the sensor implementation is applicable in the dynamic range from µT to T. To measure the sensitivity of the device at resonance, it is operated at its resonance frequency while the external magnetic field in the *z*-direction is varied from 12.6 mT to 82 mT and the differential increments in resonance amplitude corresponding to the these external magnetic fields are measured using optical microscope as described in Section 4.3. The sensitivity at resonance is found to be 1.35 µm/mT for 8 mA current as shown in Figure 10 and is compared to the theoretically calculated value of 1.29 µm/mT showing good agreement with a percentage difference of 4.6.

**Figure 11:**
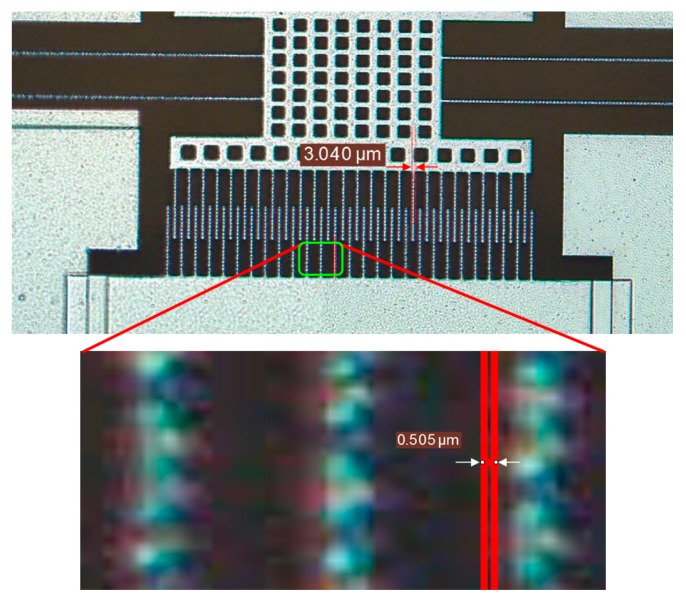
Minimum measured dimension under Leica microscope with more clear close-up view.

To determine the minimum magnetic field detectable by the device, the minimum dimension measurement (noise floor) of the optical characterization setup is determined. The measured shuttle finger width is 3 µm and the minimum measurement that can be taken with this microscope is 0.505 µm as shown in the inset of Figure 11. The resolution of the sensor is determined by taking the ratio of optical noise (0.505 µm) to the sensitivity (1.35 µm/mT) of the sensor and found to be 370.37 µT. The desk-top optical setup used in this study is to characterize the sensor parameters and will therefore not be used in field applications. For a compact and complete sensor for field applications, comb fingers are fabricated on both sides of the shuttle device for capacitive transduction.

The sensitivity and resolution will be much higher by using this capacitive transduction method and the output will be purely in electrical domain. The output from these comb fingers will be characterized in a future study.

## 6. Conclusions

This article describes the working principle, fabrication and characterization of a CMOS-MEMS resonant magnetic field sensor with a dynamic range from µT to T. The sensor is fabricated from thin layers of 0.35 µm CMOS technology and dry post-micromachining is used to release the structure. From its optical characterization, the resonance frequency of the shuttle is determined to be 8164 Hz, its quality factor to be 51.34 and damping ratio is 0.00973 when operated in air. The sensitivity of the sensor is determined in static mode as well as at resonance and the sensitivity at resonance is found to be 1.35 µm/mT. The sensitivity of the device is found to increase with increasing biasing current and the resolution of the sensor is found to be 370.37 µT.
